# Effectiveness of Endobronchial Ultrasound-Guided Transbronchial Biopsy Combined With Tissue Culture for the Diagnosis of Sputum Smear-Negative Pulmonary Tuberculosis

**DOI:** 10.3389/fmicb.2022.847479

**Published:** 2022-04-25

**Authors:** Ching-Kai Lin, Hung-Jen Fan, Kai-Lun Yu, Lih-Yu Chang, Yueh-Feng Wen, Li-Ta Keng, Chao-Chi Ho

**Affiliations:** ^1^Department of Medicine, National Taiwan University Cancer Center, Taipei City, Taiwan; ^2^Department of Internal Medicine, National Taiwan University Hospital, Taipei City, Taiwan; ^3^Department of Internal Medicine, National Taiwan University Hsin-Chu Hospital, Hsinchu, Taiwan; ^4^Department of Internal Medicine, National Taiwan University Biomedical Park Hospital, Hsinchu, Taiwan

**Keywords:** echoic feature, endobronchial ultrasound-guided transbronchial biopsy, positive tuberculosis culture rate, sputum smear-negative pulmonary tuberculosis, tissue culture

## Abstract

**Background:**

Microorganisms of tuberculosis (TB) are frequently difficult to identify from the airway specimen; therefore, lung biopsy for further histologic and microbiologic study is required. Endobronchial ultrasound-guided transbronchial biopsy (EBUS-TBB) is used for the diagnosis of pulmonary malignancy, but is rarely in the TB population. The purpose of this study was to verify the effectiveness and safety of EBUS-TBB with histologic study and tissue culture in the diagnosis of sputum smear-negative pulmonary TB.

**Methods:**

Patients who underwent EBUS-TBB with histologic study and TB tissue culture for clinically suspected, but sputum smear-negative pulmonary TB from January 2016 to December 2018, were included. The accuracy of each diagnostic modality was calculated, respectively. Factors that might influence the positive rate of TB culture (washing fluid and tissue specimen) were also evaluated.

**Results:**

One hundred sixty-one patients who underwent EBUS-TBB for clinically suspected, but sputum smear-negative pulmonary TB, were enrolled, and 43 of them were finally diagnosed as having pulmonary TB. The sensitivity of washing fluid (a combination of smear, culture, and polymerase chain reaction for TB) and tissue specimen (a combination of pathology and tissue culture) *via* EBUS-TBB for TB diagnosis were 48.8 and 55.8%, respectively. The sensitivity for TB diagnosis would be elevated to 67.4% when both washing fluid and tissue specimens are used. The positive TB culture rate would not statistically increase with a combination of tissue specimens and washing fluid. Univariate analysis revealed that TB microorganisms would be more easily cultivated when lesions had an abscess or cavity on the computed tomography (CT) image (presence vs. absence; 62.5 vs. 26.3%, *p* = 0.022), heterogeneous echogenicity on the EBUS finding (heterogeneous vs. homogeneous; 93.3 vs. 21.4%, *p* = 0.001), or a necrotic pattern *via* histologic study (presence vs. absence; 70.6 vs. 30.8%, *p* = 0.013). Heterogeneous echogenicity in the EBUS finding was the independent predictor according to the results of multivariate analysis. None of our patients encountered major adverse events or received further intensive care after EBUS-TBB.

**Conclusion:**

Endobronchial ultrasound-guided transbronchial biopsy is safe and effective for use in diagnosing sputum smear-negative pulmonary TB. EBUS echoic feature is also a predictor of the positive TB culture rate in pulmonary TB. However, tissue culture *via* EBUS-TBB has little effect in improving the positive TB culture rate.

## Introduction

Tuberculosis (TB) is one of the most prevalent infectious diseases in the world, and lung nodules or lung consolidation are common manifestations (Nachiappan et al., 2017). These representations are also frequently seen in other infectious diseases and pulmonary malignancies; therefore, identifying the microorganism is an essential step in the diagnosis of pulmonary TB and in devising an appropriate treatment plan. Analyzing a smear, culture, or polymerase chain reaction for TB (TB-PCR) of the sputum is the basic diagnostic method (Hopewell et al., 2006). Flexible bronchoscopy for extracting the distal airway specimen can be considered when the patient is unable to produce expectorate. A negative microbiologic result frequently occurs, so lung biopsy for further histologic and microbiologic study is needed (Dhamija et al., 2019; Lin et al., 2020). Besides surgical biopsy, computed tomography (CT)-guided biopsy is the traditional way to perform lung lesion sampling. Due to its high complication rate, which may lead to patient morbidity and mortality, further less invasive and safer procedures are required for confirming sputum smear-negative pulmonary TB (White et al., 2000).

Endobronchial ultrasound (EBUS), a miniature ultrasound probe that is inserted through a flexible bronchoscope to scan the bronchial lumen, was first introduced in 1990 (Hürter and Hanrath, 1992). It not only confirms the location of the pulmonary lesions but also analyze their internal structure and help pulmonologists perform transbronchial biopsy (TBB) for peripheral pulmonary lesion sampling (Kurimoto et al., 2002). Previous studies have reported the high accuracy and low complication rate of EBUS in the diagnosis of pulmonary malignancy (Herth et al., 2002; Yamada et al., 2007). However, there is scant evidence as to the utility of EBUS-guided TBB (EBUS-TBB) in the diagnosis of pulmonary TB. Lung tissue obtained *via* EBUS-TBB is seldom used for tissue culture. Therefore, our aims in this study were to investigate the effectiveness of EBUS-TBB combined with histologic study and tissue culture in the diagnosis of sputum smear-negative pulmonary TB, and to evaluate the safety of EBUS-TBB in this population of patients.

## Materials and Methods

### Participants

This was a retrospective chart review of patients who underwent EBUS-TBB for clinically suspected pulmonary TB, performed by pulmonologists or infectious disease physicians at the Department of Thoracic Medicine, National Taiwan University Hospital and National Taiwan University Hsin—Chu Hospital, from January 2016 to December 2018. Before undergoing EBUS-TBB, all patients with suspected sputum smear-negative pulmonary TB needed to have at least three negative Ziehl–Neelsen smear results from sputum analysis or those patients who were unable to produce expectorate. EBUS-TBB procedures without histologic study or tissue culture were excluded. Patients with an endobronchial lesion that was found during the bronchoscopic exam were also not included. Patient data regarding age, gender, immunocompromised status, and the final diagnosis were collected. The characteristics of the lesions (location, size, and CT image with a cavity/abscess pattern), antibacterial agent use prior to EBUS procedures, EBUS characteristics (echogenicity and probe location), pathologic findings, and procedure-related adverse events were also recorded. Written informed consent was obtained from each patient prior to bronchoscopy. The study was approved by the National Taiwan University Hospital Institutional Review Board (IRB #202109054RINB).

### Procedures

All procedures were performed by our pulmonologists, who had at least 2 years of experience in EBUS, or by our senior pulmonary fellow doctors supervised by an experienced pulmonologist. The procedure was performed with a flexible bronchoscopy (BF-Q290; Olympus Co., Tokyo, Japan) combined with a 20 MHz radial-EBUS (UM-S20-20R; Olympus Co., Tokyo, Japan). After premedication with a lidocaine local anesthesia, with or without intravenous midazolam and fentanyl for conscious sedation, the scope was passed through the nasal route. The EBUS probe was inserted through the working channel of the scope into the suspected target bronchus based on the CT image. After confirming the location of the lesion by EBUS, TBB with forceps was carried out for specimen collection. At least four biopsy sample materials were placed in 10% formalin for routine histologic evaluation. One biopsy specimen was placed in 2–3 ml of sterile normal saline for TB tissue culture. After the EBUS-TBB procedure, 25 ml of sterile normal saline was irrigated into the target bronchus, and then the washing fluid was retaken and sent to our microbiology laboratory for Gram stain, Ziehl–Neelsen smear, culture (bacterial, TB, fungal), and TB-PCR.

The diagnostic criteria of pulmonary TB were established based on pathologic evidence, microbiological analyses, or clinical follow-up. If the culture showed positive for a TB microorganism, or acid-fast bacilli or granulomatous inflammation were present in the histologic finding, or the chest CT image led the pulmonologists or infectious disease physicians to suspect a TB infection, and/or there was a response to anti-TB treatment with improvement in the image follow-up, pulmonary TB would be confirmed. Other etiologies of the pulmonary lesions were also diagnosed based on pathologic or microbiological results. When the culture was positive for bacteria, there was a response to antibacterial agent treatment with improvement in the image follow-up, and contamination or specific infections (fungal and mycobacterial)/inflammation process have been excluded, bacterial pneumonia or lung abscess would be considered.

### Statistical Analysis

In our study population, the sensitivity, specificity, positive predictive value (PPV), negative predictive value (NPV), and diagnostic accuracy of each diagnostic modality (pathology, smear, culture, and TB-PCR) were calculated, respectively, *via* standard definitions. The positive TB culture rate (tissue specimen or washing fluid) was assessed for patients who had a final diagnosis of TB infection, and the definition was “a positive result for TB culture class/total TB infection class.” Comparisons were made using Student’s *t*-test or one-way analysis of variance (ANOVA) for continuous variables, and the χ^2^ test or Fisher’s exact test for categorical variables. Logistic regression identified the independent variables contributing to statistical significance. Odds ratios (OR) and their 95% confidence intervals [95% confidence (CI)] were determined to assess the contribution of significant factors. A *p*-value of less than 0.05 was considered significant. We used SPSS version 21.0 (IBM, SPSS, Chicago, IL, United States) for statistical analysis.

## Results

### Patients and Endobronchial Ultrasound Procedures

In all, 307 patients with suspected, but sputum smear-negative pulmonary TB underwent EBUS-TBB procedures during our study period. After excluding 120 patients who had a final diagnosis of malignancy, and 26 patients who were lost to our clinical follow-up without a definite diagnosis, we finally enrolled 161 patients in our study group. About half of the study population (48.4%) was immunocompromised patients. Most of the pulmonary lesions were located at the upper lobe (34.8% in the right upper lobe and 21.1% in the left upper lobe). The mean lesion size was 40.2 mm, and 44.1% had an abscess or cavity within the lesion, as detected in the CT image. The EBUS probe arrived within the lesion in 87% of the study population, and heterogeneous echogenicity was seen in 26.1% of the lesions. Thirty-five patients received antibacterial agents before EBUS-TBB. Fifteen patients had a procedure-related adverse effect after EBUS-TBB, as follows: fever in seven patients; pneumothorax in five patients, but no need for tube drainage; bronchospasm in one patient; transient hypoxia with complete recovery in one patient; and persistent bleeding in one patient who needed instillation of a vasoactive agent and a prolonged scope wedge. None of our patients required further intensive care after the procedure (Table 1).

**TABLE 1:** Characteristics of the patients, target lesions, and endobronchial ultrasound (EBUS) procedures.

Characteristics	*N*
Number	161
Age (years old, range)	58.9 (20–93)
Male gender (%)	109 (67.7)
Immunocompromised host (%)	78 (48.4)
Hematologic disease	9 (5.6)
Solid malignancy	18 (11.2)
Other systemic disease	51 (31.7)
Diabetes mellitus with poor control	23 (14.3)
Long-term steroid use	19 (11.8)
End-stage renal disease	4 (2.5)
Liver cirrhosis	5 (3.1)
Lesion location	
Right upper lobe	56 (34.8)
Left upper lobe (left upper division and left lingual lobe)	34 (21.1)
Right middle lobe	18 (11.2)
Right lower lobe	39 (24.2)
Left lower lobe	14 (8.7)
Lesion size (mm, range)	40.2 (4–118.4)
Lesion character with abscess/cavity formation	71 (44.1)
EBUS probe location (within,%)	140 (87.0)
Echogenicity under EBUS (heterogeneous,%)	42 (26.1)
Antibacterial agent used prior to EBUS procedures	35 (21.7)
Procedure-related complications (%)	15 (9.3)
Post-procedure fever	7 (4.3)
Pneumothorax	5 (3.1)
Bronchospasm	1 (0.6)
Hypoxia	1 (0.6)
Bleeding	1 (0.6)

*EBUS, endobronchial ultrasound; N, number.*

The final diagnosis revealed that 43 of the 161 patients had pulmonary TB, 10 had non-tuberculous mycobacterial infection, 18 had fungal infection, 61 had bacterial pneumonia or lung abscess, and 11 had interstitial lung disease. The remaining 18 patients had no specific pathological or microbiological diagnosis and were classified as having non-specific inflammation (Table 2).

**TABLE 2:** Final diagnosis of the 161 patients.

Final diagnosis	*N* (%)
Mycobacterium tuberculosis	43 (26.7)
Non-tuberculous mycobacteria	10 (6.2)
*Mycobacterium avium intracellulare* complex	4 (2.5)
*Mycobacterium kansasii*	3 (1.9)
*Mycobacterium abscessus*	2 (1.2)
*Mycobacterium gordonae*	1 (0.6)
Fungus	18 (11.2)
*Candida*	2 (1.2)
*Aspergillus*	8 (5.0)
Cryptococcosis	7 (4.3)
Mucormycosis	1 (0.6)
Bacterial pneumonia/abscess	61 (37.9)
*Klebsiella pneumoniae*	32 (19.9)
*Pseudomonas aeruginosa*	12 (7.5)
*Escherichia coli*	9 (5.6)
*Enterobacter* spp.	7 (4.3)
*Mycoplasma pneumoniae*	1 (0.6)
Interstitial lung disease	11 (6.8)
Organizing pneumonia	7 (4.3)
Connective tissue disease associated with interstitial lung disease	2 (1.2)
Sarcoidosis	1 (0.6)
Pneumoconiosis	1 (0.6)
Non-specific inflammation	18 (11.2)

*N, number.*

### Performance of Each Diagnostic Modality for Tuberculosis Diagnosis

For the accuracy of the washing fluid, the Ziehl–Neelsen smear had 2.3% sensitivity, 100% specificity, 100% PPV, 73.8% NPV, and 73.9% diagnostic accuracy. Culture for TB had 41.9% sensitivity, 100% specificity, 100% PPV, 82.5% NPV, and 84.5% diagnostic accuracy. TB-PCR had 30.2% sensitivity, 99.2% specificity, 92.9% PPV, 79.6% NPV, and 80.8% diagnostic accuracy. The combination of Ziehl–Neelsen smear, culture, and TB-PCR had 48.8% sensitivity, 100% specificity, 100% PPV, 84.3% NPV, and 86.3% diagnostic accuracy (Table 3).

**TABLE 3:** Performance of diagnostic modalities for TB infection in isolation and in combination.

	Sensitivity (%)	Specificity (%)	PPV (%)	NPV (%)	Accuracy (%)
**Washing fluid study**					
Ziehl–Neelsen smear	2.3	100	100	73.8	73.9
TB culture	41.9	100	100	82.5	84.5
TB-PCR	30.2	99.2	92.9	79.6	80.8
Smear + culture + PCR	48.8	100	100	84.3	86.3
**Tissue specimen study**					
Histologic finding	41.9	91.5	64.3	81.2	78.3
TB tissue culture	27.9	100	100	79.2	80.8
Histology + tissue culture	55.8	100	100	86.1	88.2
**Fluid + tissue study**					
Washing fluid (smear + culture + PCR) + histologic finding + tissue culture	67.4	100	100	89.4	91.3

*NPV, negative predictive value; PCR, polymerase chain reaction; PPV, positive predictive value; TB, tuberculosis.*

For tissue specimen analysis, the sensitivity, specificity, PPV, NPV, and diagnostic accuracy of typical histologic findings (positive of acid-fast bacilli or granulomatous inflammation) alone were 41.9, 91.5, 64.3, 81.2, and 78.3%, respectively. The sensitivity, specificity, PPV, NPV, and diagnostic accuracy of tissue culture for TB were 27.9, 100, 100, 79.2, and 80.8%, respectively. Combining the histologic finding and tissue culture, the sensitivity, specificity, PPV, NPV, and diagnostic accuracy were 55.8, 100, 100, 86.1, and 88.2%, respectively. When washing fluid and tissue specimens were combined for the diagnosis of TB infection, the sensitivity, specificity, PPV, NPV, and diagnostic accuracy were 67.4, 100, 100, 89.4, and 91.3%, respectively (Table 3).

### Subgroup Analysis of Patients Diagnosed With Tuberculosis Infection

**FIGURE 1:**
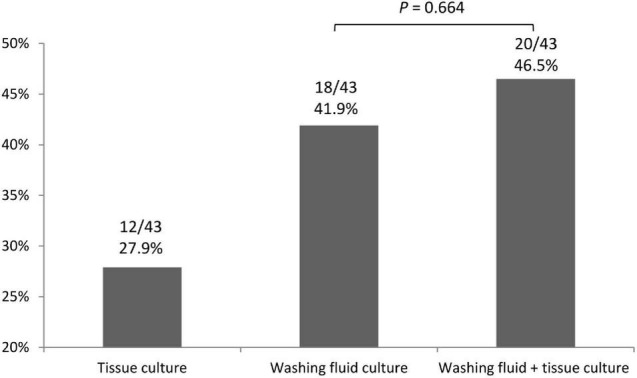
The positive TB culture rate of washing fluid and tissue specimen *via* EBUS–TBB. EBUS–TBB, endobronchial ultrasound-guided transbronchial biopsy; TB, tuberculosis.

Twenty-nine (67.4%) of the 43 patients who had a final diagnosis of TB infection were diagnosed *via* bronchoscopic exam. Only four patients had a procedure-related adverse effect after EBUS-TBB (Table 4). The positive TB culture rate of washing fluid and tissue *via* EBUS-TBB were 41.9 and 27.9%, respectively. The positive culture rate of the washing fluid did not statistically improve when combining the tissue specimen for analysis (*p* = 0.664) (Figure 1).

**TABLE 4:** Characteristics of the 43 patients with pulmonary TB infection.

Characteristics	*N*
Number	43
Age (years old, range)	54.9 (20–89)
Male gender (%)	32 (74.4)
Procedure-related complications (%)	4 (9.3)
Post-procedure fever	1 (2.3)
Pneumothorax	1 (2.3)
Bronchospasm	1 (2.3)
Bleeding	1 (2.3)
Final diagnostic modalities (%)	
Bronchoscopy	29 (67.4)
Endobronchial ultrasound guided-transbronchial needle aspiration	3 (7.0)
Computed tomography guided-biopsy	2 (4.7)
Image follow-up	9 (20.9)

*N, number; TB, tuberculosis.*

We also evaluated some factors that might influence the positive rate of TB culture when using washing fluid and tissue specimens. Univariate analysis revealed that lesions with abscess or cavity patterns on CT imaging had a higher positive TB culture rate (62.5 vs. 26.3%, *p* = 0.022). A superior positive TB culture rate was also seen when heterogeneous echogenicity was detected in the EBUS study (93.3 vs. 21.4%, *p* = 0.001). If a necrotic pattern was disclosed within the histologic specimens, microorganisms would be more easily cultivated (70.6 vs. 30.8%, *p* = 0.013). We also used abscess or cavity patterns on CT imaging, heterogeneous echogenicity on EBUS study, and necrosis within the histologic specimens for multivariate analysis. Heterogeneous echogenicity was the independent factor, with an OR of 44.33 (95% CI, 3.70–530.93). The patient’s immunocompromised status, the location, and size of the lesion, and the position of the probe did not affect the positive TB tissue culture rate *via* EBUS-TBB (Table 5).

**TABLE 5:** Logistic regression analysis of factors influencing the positive TB culture rate of washing fluid and tissue specimen.

Factors	Positive TB tissue culture rate (%)		Univariate		Multivariate	
	Factor presence	Factor absence	OR (95% CI)	*p*-Value	OR (95% CI)	*p*-Value
Patient characteristics						
Immunocompromised hosts	8/20 (40)	12/23 (52.2)	0.61 (0.18–2.05)	0.426	–	–
Lesion characteristics						
Location (upper lobe)	16/35 (45.7)	4/8 (50)	0.84 (0.18–3.92)	0.827	–	–
Size (≥ 3 cm)	13/26 (50)	7/17 (41.2)	1.43 (0.42–4.91)	0.571	–	–
Abscess/cavity patterns	15/24 (62.5)	5/19 (26.3)	6.07 (1.14–32.41)	0.022*	1.07 (0.16–7.26)	0.941
EBUS characteristics						
Heterogeneous echogenicity	14/15 (93.3)	6/28 (21.4)	51.33 (5.57–472.89)	0.001*	44.33 (3.70–530.93)	0.003*
Probe within the lesion	18/37 (48.6)	2/6 (33.3)	1.90 (0.31–11.64)	0.490	–	–
Pathologic finding						
Present necrosis	12/17 (70.6)	8/26 (30.8)	5.40 (1,42–20.52)	0.013*	4.32 (0.71–26.32)	0.113

*CI, confidence intervals; EBUS-TBB, endobronchial ultrasound-guided transbronchial biopsy; OR, odds ratio; TB, tuberculosis.*

*Statistical significance with p-value < 0.05.

## Discussion

This retrospective study showed that EBUS-TBB, with a low complication rate, improved the diagnostic accuracy of sputum smear-negative pulmonary TB. Also, when the lesions had heterogeneous echogenicity in the EBUS finding, a TB organism would be more easily cultivated.

The diagnosis of pulmonary TB infection is conventionally made *via* sputum or bronchoscopy with bronchoalveolar lavage analysis (Jacomelli et al., 2012). However, the sensitivity of bronchial washing fluid analysis seems to be insufficient for sputum smear-negative pulmonary TB. Even with a combined Ziehl–Neelsen smear, TB culture, and TB-PCR, the sensitivity was only 48.8% in our study. Tissue sampling is still essential to aid diagnostic accuracy in this population (Beck et al., 2019). An endobronchial lesion is uncommon in sputum smear-negative pulmonary TB (Jacomelli et al., 2012), therefore, some bronchoscopic techniques, such as fluoroscopy, bronchoscopic navigation, or EBUS are required to guide the peripheral lung lesion biopsy (Ishida et al., 2011; Boonsarngsuk et al., 2012; Asano et al., 2015). In the present study, we used EBUS to guide the lung biopsy. The sensitivity of tissue specimens (combined histologic and TB culture) *via* EBUS-TBB was higher than with washing fluid analysis alone. The sensitivity was also elevated to nearly 70% when both washing fluid and tissue specimens were used. We confirmed that EBUS-TBB could help increase the sensitivity for sputum smear-negative pulmonary TB diagnosis.

Image-guided procedures, such as CT-guided percutaneous lung biopsy, have a high rate of accuracy for the diagnosis of peripheral pulmonary lesions. However, pulmonary hemorrhage and pneumothorax frequently occur during the procedure, at a rate of 10-40% (White et al., 2000; Yun et al., 2018). Fatal complications, such as systemic air embolism, may even cause patient mortality (Lang et al., 2018; Sakatani et al., 2018). In the present study, less than 10% of all study participants had a procedure-related adverse effect after EBUS-TBB, and none of them needed further intensive care. Pneumothorax occurred in five patients (3.1%), and none of them required tube drainage. In Lin’s study, only one patient (1%) developed pneumothorax after the EBUS procedure (Lin et al., 2009). In another study, no adverse effect was found when combining EBUS and electromagnetic navigation for pulmonary TB diagnosis (Gu et al., 2019). Infection is also a common adverse event in many invasive procedures. However, only 4.3% of our study population had new fever episode after EBUS-TBB, and all of them recovered rapidly after a short course of antibacterial agent treatment. In our institution, prophylactic antibacterial agents are not routinely used for all bronchoscopic procedures. Only 35 patients (21.7%) in our study population used antibacterial agents prior to EBUS procedures due to persisting fever and bacterial infection could not be excluded. In addition, image-guided lung sampling can obtain specimens from a single pulmonary lesion only. Unlike bronchoscopic procedures, it is difficult to collect specimens simultaneously from the target lesion and all parts of bronchial tree. We believe EBUS-TBB is safe and has a high degree of efficacy for the diagnosis of pulmonary TB.

The TB culture result not only confirms the presence of a microorganism, but also provides information on drug sensitivity, which may guide clinicians in devising an appropriate treatment plan. Some factors were thought to affect the positive rate for TB culture *via* bronchoscopic procedures. Large lesion size or patients with an immunocompromised status might be considered to have a higher TB organism burden within the lesion. In the present study, the TB culture rate using washing fluid and tissue specimen was not statistically different in these situations. The reason might be that the TB organism is not distributed evenly. In previous studies, higher TB organism burdens were usually found in central, necrotic zones of necrotizing granulomas (Radhika et al., 1989; El-Zammar and Katzenstein, 2007). In the present study, the lesions with abscess or cavity patterns on CT imaging also had a higher positive TB culture rate. EBUS-TBB pathology showing a necrosis pattern had a significantly higher positive culture rate than those without a necrotic component (70.6 vs. 30.8%, *p* = 0.013). This result is very similar to that of our previous study, which found that a higher positive microbiologic yield will be obtained using the EBUS-TBNA procedure when the pathologic specimen shows a necrotic tissue component (Lin et al., 2020). TB organisms might be difficult to identify in external or non-necrotic parts of the lesion. Finding a proper site might influence the probability to expose TB organism in the diagnosis of pulmonary TB during bronchoscopy exam.

Tuberculosis organisms would be easier to cultivate when the lung lesions show heterogeneous echogenicity *via* the EBUS image, as in our study result. Heterogeneous echogenicity means that non-unique components, such as necrosis or cavitation, exist in the structure of the lung lesions (Kuo et al., 2011). As such, necrotic components are likely to be obtained in that part of the lung lesion with heterogeneous echogenicity, which retains a higher TB bacterial burden. In the present study, heterogeneous echogenicity in the EBUS finding was the only independent predictor according to the results of multivariate analysis (*p* = 0.003; OR of 44.33; 95% CI, 3.70–530.93). This result shows that the part of the lesion with heterogeneous echogenicity in the EBUS findings should be the better site for discovering TB organisms. To our knowledge, this was the first study to confirm the echoic feature as a predictor of the positive TB culture rate in pulmonary TB. We can even use the EBUS image pattern to guide us to a better site to diagnose pulmonary TB.

Apart from using specimens from the respiratory tract for TB culture, tissue culture *via* EBUS-TBB was also used for pulmonary TB diagnosis in our study. In previous literatures, tissue specimens obtained *via* image-guided percutaneous lung biopsy used for TB tissue culture has improved the diagnosis of pulmonary TB (Park et al., 2010; Zhao et al., 2020). However, there are a few reports on using tissue specimens *via* TBB for TB culture. Several studies have revealed that specimens obtained *via* bronchial washing were more sensitive for culture positivity than specimens obtained *via* TBB; therefore, using TBB for bacterial investigations is of limited diagnostic value (Chan et al., 1992; Sekine et al., 2017). Unlike the traditional transbronchial lung sampling method, which does not use a guiding system in the previous study, utilizing EBUS can help confirm the location of the lesions, and assist the operators in choosing a better site for biopsy. We performed EBUS-TBB for tissue culture, but the positive culture rate of TB tissue culture was relatively low. The positive culture rate of the washing fluid did not statistically improve when combining tissue specimens for analysis (*p* = 0.664). Tissue culture *via* EBUS-TBB has little effect on improving the positive TB culture rate. Moreover, the sensitivity of TB tissue culture alone was even lower than histologic findings *via* EBUS-TBB. It seems that defining granulomatous inflammation from histologic examination, but not tissue TB culture, is the main merit of the EBUS-TBB approach for diagnosing pulmonary TB. Nevertheless, two patients (4.7%) in our TB population had positive TB tissue culture results with negative washing fluid culture. In these circumstances, we believe that tissue culture is still required for clinicians to assist with pulmonary TB treatment.

Our study, however, has several limitations. First, this was a retrospective study, so there might be a risk of selection bias in our study population. For example, patients who did not undergo EBUS-TBB and those for whom specimens were not obtained for tissue culture were not enrolled. Second, the sample size was relatively small. Some special population groups, such as human immunodeficiency virus-infected hosts, who are also at risk of TB infection, were not enrolled. Although rare serious complications such as severe infection, abscess formation, hemodynamic changes, or acute respiratory events were not seen in our small study population, we are unable to determine whether these complications would not occur when the sample size is increased. Third, we did not perform brushing or use guide sheath in our institution when pulmonary infection was suspected because extra cost was needed. Previous publications revealed that TBB combined with these techniques has higher sensitivity for the diagnosis of peripheral pulmonary lesions (Kovnat et al., 1975; Kikuchi et al., 2004; Kurimoto et al., 2004), so they are performed routinely in many institutions. Therefore, a prospective design with a comprehensive study group and various diagnostic devices is warranted. Finally, not all of our TB patients had a diagnosis based on pathologic or microbiologic evidence. Nine patients (20.9%) had the TB diagnosis based on the clinical criteria. However, all of these nine patients had typical CT image pattern and the image improved after completing the anti-TB treatment course without any antibacterial agent. In endemic areas, clinical physicians consider TB infection if a typical pathologic pattern, positive microbiologic report (Ziehl-Neelsen smear, culture, and TB-PCR), or even a typical CT image pattern is presented. We also used the image response after a complete anti-TB treatment course. We think our results are more easily applied in clinical practice.

In conclusion, EBUS-TBB improves the diagnosis of pulmonary TB. The TB organism is easier to detect in the necrotic part of the lesion; therefore, lesions with abscess/cavity patterns or heterogeneous echogenicity on EBUS findings had a higher positive TB culture rate. We believe that the EBUS image pattern with heterogeneous echogenicity can be used for guidance of TBB and collecting airway specimens when TB infection is highly suspected. However, tissue culture *via* EBUS-TBB has little effect on improving the positive TB culture rate.

## Data Availability Statement

The original contributions presented in the study are included in the article/supplementary material, further inquiries can be directed to the corresponding author.

## Ethics Statement

The studies involving human participants were reviewed and approved by the study was approved by the National Taiwan University Hospital Institutional Review Board (IRB #202109054RINB). The patients/participants provided their written informed consent to participate in this study.

## Author Contributions

C-KL and C-CH contributed to the study concept and design, acquisition, analysis, interpretation of data, and drafting of the manuscript. C-KL, H-JF, K-LY, L-YC, Y-FW, and L-TK contributed to the data collection and manuscript review. C-KL, Y-FW, and C-CH contributed to the study concept and design, analysis and interpretation of data, and critical revision of the manuscript for important intellectual content. C-CH supervised the study. All authors read and approved the final manuscript.

## Conflict of Interest

The authors declare that the research was conducted in the absence of any commercial or financial relationships that could be construed as a potential conflict of interest.

## Publisher’s Note

All claims expressed in this article are solely those of the authors and do not necessarily represent those of their affiliated organizations, or those of the publisher, the editors and the reviewers. Any product that may be evaluated in this article, or claim that may be made by its manufacturer, is not guaranteed or endorsed by the publisher.
